# Gender differences and occupational factors for the risk of obesity in the Italian working population

**DOI:** 10.1186/s12889-020-08817-z

**Published:** 2020-05-16

**Authors:** C. Di Tecco, L. Fontana, G. Adamo, M. Petyx, S. Iavicoli

**Affiliations:** 1 Italian Workers’ Compensation Authority (INAIL), Department of Occupational and Environmental Medicine, Epidemiology and Hygiene, Monte Porzio Catone, 00078 Rome, Italy; 2 Department of Public Health and Infectious Diseases, Sapienza University of Rome, Rome, Italy

**Keywords:** Sex differences, Body mass index, Overweight, Work-related aspects, Work shift, Occupational sectors

## Abstract

**Background:**

Obesity is a multifactorial condition and a major risk factor associated with several non-communicable diseases, such as cardiovascular disease, and with a higher risk of premature death and disability. Sex-specific factors have key roles and must be taken into consideration in studying occupational factors associated with the risk of obesity. The aim of this study was to investigate gender differences in body mass index (BMI) in a large cohort representative of Italian workers and, correlating this index with several demographic and occupational variables, to verify sex- and work-dependent differences in the risk of obesity.

**Methods:**

We utilized data from INSuLa, a cross-sectional, nationally representative survey of the Italian worker population conducted in 2013 by the Italian Workers’ Compensation Authority to investigate health and safety at work. Analyses were run on a sample of 8000 Italian workers, aged from 16 to 64 years. Logistic regression models were employed to assess gender differences in the relation between occupational characteristics and BMI. We adjusted for age, education, variables related to health protection at work, and chronic conditions and diseases.

**Results:**

There were several significant differences in the BMI between males and females, linked to some occupational factors. For instance, female shift workers were 1.32 times (95% CI 1.11–1.57) more likely to be overweight or obese than normal-weight workers, and this association was maintained when controlling for confounders. The likelihood of overweight or obesity among women who worked 1–2 night shifts per week was significantly higher – 1.5-1.6 times – than those on day shifts.

**Conclusions:**

Gender-specific differences in occupational factors associated with the risk of obesity are useful with a view to characterizing this risk and helping identify workplace-targeted intervention strategies.

## Background

The World Health Organization (WHO) defines obesity as a condition of “*abnormal or excessive fat accumulation in adipose tissue to the extent that health may be impaired*” [1, 2]. This is a complicated, multifactorial condition that should be considered a disease in its own right but it is also a major risk factor associated with several non-communicable diseases and with a higher risk of premature death and disability [2–9]. In recent decades the prevalence of obesity (and overweight) has steadily grown at an alarming rate, so that nowadays we are openly talking about a pandemic of obesity [4, 10, 11]. The most recent estimates provided by the WHO Global Health Observatory indicate that nearly two billion adults worldwide are overweight and more than half a billion of these are obese [12]. The global prevalence of obesity is higher in women than men in all continents, in both developed and developing countries [13–15].

In Italy the prevalence of obesity is 20% for both sexes, and the percentages of overweight subjects are 65% in men and 52% in women [16]. Although the current prevalence of obesity in Italy is in fact somewhat lower than in many other countries, in the last 40 years the obese population has constantly risen from approximately 7% in the 1970s to the current 20% [16]. Obviously, this has significant consequences both in terms of health and economic burden, since the reduction in life expectancy attributable to overweight is 2.7 years and obesity/overweight accounts for 9% of national health costs [16]. In addition, the labor market is significantly affected by the negative effects of obesity and the reduction in the workforce due to this condition amounts to 571,000 full-time workers per year [16].

The pathogenesis of obesity is substantially correlated to a long-term energy imbalance between too many calories consumed and too few calories expended, that can result from a combination of over-eating, scant energy expenditure, and physical inactivity [17]. Each of these parameters is strongly influenced by a myriad of demographic, social, cultural and occupational factors [14, 17]. Many such variables probably concur in increasing obesity in women and some crucial aspects of metabolic homeostasis may affect the onset of obesity in a substantially different manner in the two sexes, since they are regulated differently in males and females [13]. Fundamental sex differences include distribution and mobilization of adipose tissue storage, different insulin sensitivity and lipoprotein profiles, and effects of gonadal hormones [18, 19]. Therefore sex-specific biological factors play a key role in the etiopathogenesis and must be taken into consideration when studying occupational factors associated with the risk of obesity.

Clearly individual physiology and behavior can also be affected by socio-cultural and environmental determinants [14]. In other words, certain sex-specific factors predispose to obesity, which in turn may develop into specific obesogenic settings. All this information gives a fairly accurate picture of the incredible complexity of the possible causal factors and their interplays behind obesity.

Workplaces should be considered in all respects potential obesogenic environments since they can directly affect weight-related behaviors and the lifestyles of workers while at the same time exposing them to occupational risk factors that can have a significant impact on the physiology of the human body [20–23]. For example, scientific progress and technological innovation have greatly influenced the labor market, leading to increased levels of computerization, automation and mechanization [21, 24, 25]. These changes have modified the types of task in which workers are involved, reducing strenuous heavy work while increasing sedentary jobs [26–28]. This trend is likely to be confirmed (and may possibly even increase) in coming years by the so-called “fourth industrial revolution” or Industry 4.0 [29]. One must also bear in mind that adults of working age spend as much as 50% of their days (for approximately 40 h per week) in the workplace [30, 31]. Therefore, considering that most workers eat at least one of their daily meals at work, the quality of food or the time and facilities for meals that companies offer contribute substantially to the daily energy balance, also considering that spending most of one’s waking hours at work inevitably reduces the possibility of engaging in physical activity [30].

It is not surprising therefore that different research groups have investigated the prevalence of overweight and obesity among several occupational groups and sectors in representative samples of the working population, mainly employing the body mass index (BMI). This is a simple, widely used index calculated using a person’s weight and height that classifies underweight, overweight and obesity in adults. It is one of the most helpful tools for establishing the prevalence of obesity in a population [2]. For example, Caban et al. [32], using the BMI in 603,139 United States workers belonging to 41 occupational groups, reported higher obesity rates in workers employed as motor vehicle operators, police or firefighters and other protective service workers. A similar study, in an Australian worker population (25,900 people), showed higher percentages of obesity in advanced clerical, intermediate production and transport workers and laborers [30]. Comparable findings indicating high BMI and obesity prevalence especially in transportation, commercial and protective services, health care support, welfare work and retail, wholesale trade and office workers, were reported by Proper and Hildebrand [31] and Birdsey and Sussel [20] in Dutch and US workers.

Other studies have looked further into this topic, seeking correlations between the obesity prevalence and some specific occupational factors or work organization characteristics. Associations were found with working hours, shift work, hostile work environment, sedentary work and low physical demand, company size [21, 22, 33–39].

Some of these studies also showed gender divergence in the effects of work-related factors on obesity but in most cases these results were not thoroughly analysed or discussed, and in fact several authors agree on the need for more in-depth studies to clarify the causes of the weight disparity between male and female workers [20, 30].

This is an important and timely topic especially considering the growing participation of women in the labor force and the fact that female workers are increasingly entering previously male-dominated professions and occupations [21]. Therefore the present study investigated the BMI in a large cohort representative of Italian workers, correlating this index with several demographic and work-related variables to verify the presence of gender differences in the risk of obesity. The potential effects of some work-related variables in people with weight over the normal range were investigated: these include the business sector, occupational position, type of contract, shift work, night work, working hours and the size of the firm. The purpose of the study is to investigate whether the risk of overweight could be a consequence of exposure to certain occupational or work-related factors, accounting for the differences in male and female workers, by controlling for some socio-demographic determinants.

## Methods ***

### Study population and survey procedure

This study was based on data from INSuLa, a cross-sectional nationally representative survey of the Italian workers population conducted in 2013 by the Italian Workers’ Compensation Authority (INAIL) to investigate the health and safety at work. INSuLa counts a sample of 8000 Italian workers that are representative of the entire national workforce aged from 16 to 64 years according to the sampling stratification criteria applied. Sampling was done starting from the national workforce identified by the 2012 national Labour Force Survey (excluding self-employed, military and civil protection personnel). This survey provided information to depict the country as a whole and stratify the sample based on region, workers’ sex and age, type of contract, occupational level and occupational sector, thanks to the collaboration of the Italian Institute for Statistics (ISTAT).

The INSuLa survey was conducted in the period from July to December 2013 and data were collected through structured interviews, using the Computer-assisted telephone interviewing (CATI) method, by trained interviewers from TNS Italia. A random procedure was applied to contact eligible persons by telephone (mobile and landline) and participants were selected proportionally based on the sampling strategy to match the sample characteristics.

The standardized questionnaire for the interviews was developed for the national survey INSuLa [40] and it is based on a literature review and benchmarking analysis of the main European surveys in the field. It includes several questions to investigate the main aspects linked to health and safety at work in terms of working conditions, risk exposure and perceptions, health status and outcomes, management and prevention, occupational health and safety professionals and legal aspects. Some of measures included in the questionnaire have been used in further published secondary analysis study [41, 42]. In this study, we included the main demographic and occupational variables traditionally linked to the BMI, and self-reported height and weight for computing the BMI, that have been not used elsewhere in other published studies.

### Demographic variables and health-related characteristics

Information collected by the participants included sex, age in years and education (lower and middle school, high school, university graduate and post-graduate). Some information related to health were collected, asking the participants whether they had any diseases and/or chronic conditions generally linked to overweight, such as musculoskeletal, respiratory, gastrointestinal, cardiovascular diseases and insomnia. Participants were also asked to report their height in cm and weight in kg, from which their BMI was calculated as weight in kilograms divided by the square of the height in metres (kg/m^2^). Since we were interested in analysing the potential effects of some work-related variables in people with weight over the normal range according to the WHO,^1^ we set the cut-off for overweight as BMI ≥ 25.0 kg/m2. Descriptive statistics for mean BMI (+/− SD) and prevalence of population overweight (BMI ≥25.0 kg/m2) or obesity (BMI ≥30.0 kg/m2) were tabulated by all the demographic and occupational variables included in the study and divided for sex. Logistic regression models were carried out to assess associations between occupational characteristics and BMI.

### Work-related variables

Some occupational characteristics reported by the participants were included in this study to investigate possible effects on BMI. The occupational sector was based on the nine categories from the National industrial classification of all economic activities (ATECO), the occupational position (top and middle manager, white collar, blue collar, apprentice or other), the type of contract (permanent, fixed-term or temporary), shift work (yes/no), night work (none, 1 to 2 times a week, more than 2 times a week), working hours (usual number of hours worked per week in the last 6 months); the size of the firm (4 categories 1 to 9 employees, 10–49 employees, 50–249 employees and more than 250 employees). We also included some information related to health and safety protection at work such as being included in health surveillance (yes/no), exposure to Video Display Terminals (VDT; yes/no) and work-related stress risk (yes/no). These work risks were included on account of the recognized link to the risk of overweight and obesity. Exposure to VDT was included as an indicator of sedentary work related both to musculoskeletal discomfort and the increased likelihood of sedentary behavior and reduced physical activity.

### Statistical analysis

Although the distribution of the participants was almost balanced, weighting was applied in data analysis to reach the exact proportions and reflect the population at the time of the survey, taking into account the probability of being sampled and the differences in responses. BMI was computed by dividing weight in kilograms by height in meters squared. Descriptive statistics for mean BMI (SD) and the prevalences of overweight (BMI ≥25.0 kg/m^2^) and obesity (BMI ≥30.0 kg/m^2^) were tabulated in relation to all the demographic and occupational variables, separately by sex.

Differences in BMI by occupation type were investigated by logistic regressions using BMI as dichotomous variable, taking overweight (BMI ≥25.0 kg/m^2^) as cut-off. To depict all the occupational characteristics that can be involved in BMI differences, we modelled the differences by simple regressions adjusted for age, education, variables related to health and safety protection at work (health surveillance, risk to VDT, and work-related stress risk) and chronic conditions and diseases. Analyses were stratified by sex. Occupational characteristics were entered as dummy variables and each category with the lowest mean BMI, separately for men and women, was designated the reference in all cases for ease of comparability.

Analysis was conducted in six steps: Model 1 not adjusted, Model 2 adjusted for age, Model 3 adjusted for age and education, Model 4 adjusted for age and education, exposure to VDT and health surveillance, Model 5 adjusted for age and education, exposure to VDT, health surveillance and exposure to work-related stress risk, Model 6 adjusted for age and education, exposure to VDT, health surveillance, exposure to work-related stress risk and diseases and chronic conditions. The *p* values were considered significant at *p* < 0.05 with 95% confidence interval (CI). To check residuals and verify the presence of outliers, we ran common tests in logistic regressions, namely the Hosmer-Lemeshow test and the casewise listing of residuals. Each model run showed good fit and a small number of outliers (less than 5%). All analyses were done using the IBM SPSS Statistics version 21.

## Results

Table 1 summarizes the sample, presenting the means for continuous variable and showing the percentages for categorical variables of socio-demographic and occupational features in relation to the BMI. Descriptives related to the BMI were compared by sex. The mean age of respondents was 42.7 (±9.9) years, 53.8% were men, and 49.3% had a high school education. As regard occupational characteristics, 23.2% of the sample was employed in manufacturing/industry, followed by 17.7% in commerce, 14.9% in education and public administration and 11.7% in other sectors. As regards the position held, 46.8% of respondents were blue collars, 41% white collars, 8.9% top/middle managers; almost 85% of the sample had a permanent contract. Thirty-three percent of respondents were shift workers; 5.2% had worked 1 to 2 night shifts during the last week of work, and only 3.3% had worked more than two night shifts. Sixty-three percent of the sample had worked between 35 and 40 h per week in the 6 months preceding the survey; almost 38% worked in companies with ≥250 employees.

**Table 1:** Weight status by socio-demographic, occupational and health-related characteristics, among men and women in a representative sample of 8000 Italian workers

	Males				Females			
	No.	Mean BMI (sd)	Overweight	Obese	No.	Mean BMI (sd)	Overweight	Obese
			(%)	(%)			(%)	(%)
Age group								
16–24	243	25.4 (3.8) ^a^	38.3	5.8	182	22.3 (3.4) ^a^	18.1	1.6
25–34	897	25.8 (2.9) ^ab^	48.6	7.6	752	23.0 (3.9) ^b^	20.9	3.9
35–44	1328	26.3 (3.2) ^bc^	51.2	10.0	1163	23.3 (3.8) ^bc^	19.0	5.9
45–54	1256	26.7 (3.3) ^c^	52.5	12.7	1105	24.0 (4.0) ^cd^	23.9	8.1
55–64	581	27.2 (3.8) ^d^	59.7	15.0	493	24.5 (3.7) ^d^	31.0	6.9
Education								
Lower or middle school	1558	26.7 (3.3) ^c^	55.1	12.5	904	24.4 (4.1) ^c^	29.2	8.5
High school	2039	26.3 (3.3) ^b^	50.6	10.6	889	23.4 (3.8) ^b^	19.7	3.5
University graduate and post-graduate	648	25.8 (2.9) ^a^	47.1	4.7	3647	22.9 (3.4) ^a^	22.6	6.1
Occupational sector								
Agriculture, fishing, and hunting	131	26.5 (3.1) ^ab^	52.7	12.2	55	25.0 (4.4) ^b^	36.4	7.3
Manufacturing/Primary industry/Mining/ Utilities	1356	26.4 (3.3) ^ab^	50.7	11.2	500	23.3 (3.6) ^a^	20.0	5.4
Construction	409	26.3 (2.9) ^ab^	54.1	9.0	35	23.1 (3.9) ^a^	8.6	8.6
Wholesale and retail trade/ Automotive and motorcycle repair/ Accommodation and food services	695	26.2 (3.3) ^a^	49.2	9.4	719	23.5 (4.5) ^a^	23.4	5.7
Transportation and warehousing / Information and communication	477	26.3 (3.3) ^ab^	51.4	10.5	157	23.7 (3.9) ^ab^	21.7	7.0
Professional, financial and business services	421	26.1 (3.1) ^a^	50.8	9.0	516	23.2 (3.6) ^a^	19.8	4.8
Healthcare and social assistance	194	27.1 (4.7) ^b^	56.2	16.0	505	23.8 (3.9) ^ab^	21.8	8.5
Education services/ Public administration, social security	463	26.6 (3.3) ^ab^	52.3	12.1	731	23.6 (3.6) ^ab^	22.3	5.6
Other public and personal services	159	26.3 (2.9) ^ab^	54.1	10.7	479	23.9 (3.7) ^ab^	26.7	6.3
Occupational position								
Top and middle manager	430	26.1 (3.3)	50.2	8.1	284	23.2 (3.2) ^a^	22.2	3.9
White collar	1342	26.3 (3.4)	51.4	10.3	1936	23.3 (3.6) ^a^	20.2	5.0
Blue collar	2406	26.5 (3.3)	52.1	11.5	1339	24.1 (4.3) ^b^	25.5	8.0
Apprentice or other type of employment	127	26.1 (3.5)	44.1	11.0	138	23.5 (3.8) ^ab^	23.9	7.2
Type of contract								
Permanent	3683	26.4 (3.2)	52.8	10.8	3061	23.6 (3.9)	21.3	6.5
Temporary	597	26.2 (3.8)	43.9	10.4	611	23.5 (3.6)	28.2	3.8
Shift work								
Yes	1397	26.5 (3.4)	51.4	11.7	1278	23.8 (4.1) ^f^	24.6	7.7
No	2908	26.3 (3.3)	51.5	10.3	2418	23.5 (3.8) ^f^	21.3	5.2
Night shifts								
Never	3837	26.3 (3.2)	51.0	10.6	3486	23.5 (3.9)	22.1	5.9
1 to 2 times/wk	267	26.7 (4.1)	58.4	10.5	145	24.3 (4.5)	24.1	10.3
> 2 times/wk	200	26.9 (3.6)	51.5	13.5	64	24.1 (3.4)	35.9	3.1
Working hours								
1–34 h/wk	454	26.7 (3.7) ^b^	50	13.0	1405	23.5 (3.7)	22.5	5.3
35–40 h/wk	3024	26.4 (3.3) ^ab^	51.8	10.7	2013	23.7 (4.0)	22.0	6.9
41–48 h/wk	492	26.3 (3.1) ^ab^	52.4	10.6	184	23.2 (3.5)	26.1	3.3
49–54 h/wk	202	25.9 (2.7) ^a^	49.5	6.4	59	23.2 (3.5)	25.9	1.7
> =55 h h/wk	134	26.3 (3.2) ^ab^	47.8	11.9	34	23.8 (4.5)	17.6	8.8
Firm size (no. of employees)								
1–9	651	26.2 (3.3)	47.3	10.8	665	23.2 (3.7)	23.3	4.4
10–49	887	26.2 (3.0)	54.0	8.8	727	23.6 (3.8)	22.1	5.8
50–249	892	26.4 (3.4)	51.8	11.3	796	23.9 (3.9)	25.4	7.0
≥ 250	1706	26.5 (3.2)	52.8	11.6	1313	23.5 (3.8)	20.9	6.7
Health surveillance								
Yes	3201	26.4 (3.3)	52.5	11.0	2219	23.6 (3.9) ^f^	21.9	6.9
No	322	26.5 (3.9)	45.3	12.7	408	23.2 (4.0) ^f^	19.6	4.9
Exposure to work-related stress risk								
No	2514	26.3 (3.3)	51.4	10.6	1922	23.5 (3.7)	22.6	5.6
Yes	1792	26.4 (3.3)	51.6	11.0	1774	23.6 (4.0)	22.3	6,6
Exposure to VDT								
Yes	473	26.4 (3.5)	55.2	10,4	319	23.1 (3.6) ^f^	17.2	4,7
No	3832	26.4 (3.3)	51.0	10,8	3376	23.6 (3.9) ^f^	22.9	6,2
Musculoskeletal diseases								
No	1567	25.9 (2.9) ^g^	47.5	8.2	986	23.0 (3.7) ^g^	18.7	3.3
Yes	2734	26.6 (3.5) ^g^	53.7	12.2	2705	23.8 (3.9) ^g^	23.8	7.1
Respiratory diseases								
No	4038	26.3 (3.3) ^g^	51.5	10.2	3347	23.5 (3.8) ^g^	22.6	5.3
Yes	266	27.4 (4.1) ^g^	51.1	19.2	344	24.4 (4.4) ^g^	20.6	13.7
Gastrointestinal diseases								
No	3414	26.4 (3.2)	52.3	10.6	2604	23.5 (3.8)	22.8	5.8
Yes	887	26.4 (3.8)	48.6	11.3	1084	23.6 (4.1)	21.6	6.5
Cardiovascular diseases								
No	4012	26.2 (3.2) ^g^	50.9	9.9	3409	23.4 (3.8) ^g^	21.9	5.5
Yes	292	28.1 (4.2) ^g^	58.6	22.6	280	25.2 (4.6) ^g^	29.3	13.2
Insomnia								
No	3373	26.3 (3.1) ^g^	51.5	10.2	2611	23.5 (3.9)	22.4	5.8
Yes	932	26.6 (4.0) ^g^	51.3	12.9	1080	23.7 (3.9)	22.5	6.7

Note: mean BMI for men is 26.4 (3.3); mean BMI for women 23.6 (3.9) (p < 0.05). ^a, b, c, d^ Tukey’s honestly significant difference test; ^e^ video display terminal; ^f^*p* < 0.05; ^g^*p* < 0.01

With regard to health and safety protection at work, 67.8% of the respondents received health surveillance because of exposure to a risk for health and safety at work; 90.1% reported exposure to VDT, and 55.4% exposure to work-related stress risks (sociodemographic and occupational characteristics of the sample are reported as supplemental materials).

Table 1 shows the descriptive statistics for BMI and prevalence of overweight and obesity by socio-demographic, occupational and health-related characteristics. First, the mean BMI of the Italian workers was 25.1 kg/m^2^ with 38.1% overweight and 8.6% obese (data not shown in Table 1). Men had a significantly higher mean BMI (26.4 kg/m^2^) than women (23.6 kg/m^2^) and values rose with age for both sexes. As regards education, both males and females presented a gradient, with mean BMI rising from the low to the high levels of education.

As regards the occupational sector, the BMI of male workers exceeded the normal range in all sectors, with highst levels in healthcare (27.1 kg/m^2^; 56.2% overweight and 16.0% obesity). Females working in agriculture had the highest BMI (25.0 kg/m^2^; prevalence of overweight: 36.4%).

There was a gradient from top/middle management to blue collar positions for both sexes, the latter having the highest mean BMI (26.5 men and 24.1 kg/m^2^ women). However, a significant difference was found only for women. Female shift workers had significantly higher BMI, while there were significant differences for working hours among men. No significant differences were found for the type of contract, night shift or size of the firm.

Findings of the multivariate logistic regression modelling are set out from Tables 2, 3, 4 and 5. The most significant findings are presented here.

**Table 2:** Logistic regression analyses for overweight/obesity by occupational sector, for men and women in a representative sample of 8000 Italian workers

Occupational sector	Model 1 ^a^		Model 2 ^b^		Model 3 ^c^		Model 4 ^d^		Model 5 ^e^		Model 6 ^f^	
	OR (95% CI)		OR (95% CI)		OR (95% CI)		OR (95% CI)		OR (95% CI)		OR (95% CI)	
**Males**												
Professional, financial and business services	Ref		Ref		Ref		Ref		Ref		Ref	
Agriculture, fishing, and hunting	1.26	(0.77–2.07)	1.27	(0.77–2.09)	1.11	(0.67–1.84)	1.11	(0.67–1.84)	1.11	(0.67–1.84)	1.10	(0.66–1.84)
Manufacturing/Primary industry/Mining/ Utilities	1.11	(0.85–1.45)	1.11	(0.85–1.47)	0.92	(0.70–1.23)	0.92	(0.69–1.23)	0.92	(0.69–1.23)	0.92	(0.69–1.23)
Construction	1.26	(0.90–1.76)	1.39	(0.98–1.95)	1.11	(0.78–1.57)	1.10	(0.77–1.58)	1.11	(0.77–1.58)	1.10	(0.77–1.58)
Wholesale and retail trade/Automotive and motorcycle repair/Accommodation and food services	1.13	(0.83–1.54)	1.24	(0.91–1.69)	1.03	(0.75–1.42)	1.03	(0.74–1.42)	1.03	(0.74–1.42)	1.04	(0.75–1.44)
Transportation and warehousing/ Information and communication	0.94	(0.68–1.30)	0.91	(0.66–1.26)	0.81	(0.58–1.13)	0.81	(0.58–1.13)	0.80	(0.57–1.12)	0.81	(0.58–1.14)
Healthcare and social assistance	1.56 ^g^	(1.02–2.37)	1.35	(0.88–2.08)	1.50	(0.97–2.30)	1.49	(0.96–2.31)	1.46	(0.94–2.26)	1.45	(0.93–2.26)
Education services/Public administration, social security	1.20	(0.85–1.70)	0.96	(0.67–1.37)	0.98	(0.69–1.40)	0.98	(0.68–1.40)	0.98	(0.68–1.40)	0.95	(0.66–1.36)
Other public and personal services	1.05	(0.66–1.67)	1.14	(0.71–1.82)	1.05	(0.65–1.68)	1.04	(0.65–1.68)	1.04	(0.65–1.67)	1.04	(0.65–1.67)
**Females**												
Construction	Ref		Ref		Ref		Ref		Ref		Ref	
Agriculture, fishing, and hunting	4.41 ^g^	(1.37–14.24)	4.17 ^g^	(1.28–13.58)	3.81 ^g^	(1.17–12.45)	3.40 ^g^	(1.04–11.13)	3.37 ^g^	(1.03–11.07)	3.47 ^g^	(1.05–11.50)
Manufacturing/Primary industry/Mining/ Utilities	2.05	(0.75–5.56)	2.09	(0.77–5.71)	2.05	(0.75–5.59)	1.86	(0.68–5.10)	1.85	(0.67–5.08)	1.92	(0.69–5.31)
Wholesale and retail trade/ Automotive and motorcycle repair/Accommodation and food services	2.32	(0.86–6.29)	2.49	(0.91–6.78)	2.44	(0.89–6.67)	2.25	(0.82–6.15)	2.24	(0.82–6.12)	2.24	(0.81–6.18)
Transportation and warehousing/ Information and communication	2.54	(0.90–7.22)	2.78	(0.97–7.94)	3.04 ^g^	(1.06–8.72)	2.93 ^g^	(1.02–8.40)	2.91 ^g^	(1.01–8.37)	2.96 ^g^	(1.02–8.57)
Professional, financial and business services	2.16	(0.79–5.89)	2.26	(0.82–6.22)	2.50	(0.91–6.89)	2.46	(0.89–6.78)	2.45	(0.89–6.75)	2.52	(0.91–7.01)
Healthcare and social assistance	2.63	(0.97–7.11)	2.45	(0.90–6.68)	2.87 ^g^	(1.05–7.85)	2.61	(0.95–7.15)	2.58	(0.94–7.10)	2.58	(0.93–7.15)
Education services/Public administration, social security	2.48	(0.91–6.75)	2.17	(0.79–5.95)	2.62	(0.95–7.21)	2.50	(0.91–6.88)	2.48	(0.90–6.85)	2.48	(0.89–6.91)
Other public and personal services	2.78 ^g^	(1.02–7.57)	2.91 ^g^	(1.06–7.98)	3.07 ^g^	(1.12–8.45)	2.84 ^g^	(1.03–7.82)	2.82 ^g^	(1.02–7.78)	2.85^g^	(1.02–7.91)

^a^Unadjusted; ^b^ adjusted for age; ^c^ adjusted for age, education; ^d^ adjusted for age, education, exposure to video display terminal (VDT) and health surveillance; ^e^ adjusted for age, education, exposure to VDT and health surveillance, exposure to work-related stress risk; ^f^ adjusted for age, education, exposure to VDT and health surveillance, exposure to work-related stress risk, diseases and chronic conditions; ^g^ = p < 0.05; ^h^ = p < 0.01

**Table 3:** Logistic regression analyses for overweight/obesity by type of contract, for men and women in a representative sample of 8000 Italian workers

Type of contract	Model 1 ^a^		Model 2 ^b^		Model 3 ^c^		Model 4 ^d^		Model 5 ^e^		Model 6 ^f^	
	OR (95% CI)		OR (95% CI)		OR (95% CI)		OR (95% CI)		OR (95% CI)		OR (95% CI)	
**Males**												
Permanent	1.52 ^h^	(1.23–1.88)	1.22	(0.98–1.52)	1.24	(0.99–1.54)	1.27 ^g^	(1.01–1.59)	1.27 ^g^	(1.01–1.59)	1.27 ^g^	(1.01–1.60)
Temporary	Ref		Ref		Ref		Ref		Ref		Ref	
**Females**												
Permanent	0.82	(0.65–1.03)	0.69 ^h^	(0.54–0.88)	0.71^h^	(0.56–0.91)	0.71^h^	(0.56–0.91)	0.71 ^h^	(0.56–0.91)	0.70 ^h^	(0.55–0.90)
Temporary	Ref		Ref		Ref		Ref		Ref		Ref	

^a^Unadjusted; ^b^ adjusted for age; ^c^ adjusted for age, education; ^d^ adjusted for age, education, exposure to video display terminal (VDT) and health surveillance; ^e^ adjusted for age, education, exposure to VDT and health surveillance, exposure to work-related stress risk; ^f^ adjusted for age, education, exposure to VDT and health surveillance, exposure to work-related stress risk, diseases and chronic conditions; ^g^ = p < 0.05; ^h^ = p < 0.01

**Table 4:** Logistic regression analyses for overweight/obesity by shift work, for men and women in a representative sample of 8000 Italian workers

Shift work	Model 1 ^a^		Model 2 ^b^		Model 3 ^c^		Model 4 ^d^		Model 5 ^e^		Model 6 ^f^	
	OR (95% CI)		OR (95% CI)		OR (95% CI)		OR (95% CI)		OR (95% CI)		OR (95% CI)	
**Males**												
Yes	1.16	(0.99–1.36)	1.25 ^h^	(1.06–1.46)	1.19 ^g^	(1.01–1.40)	1.19 ^g^	(1.01–1.40)	1.18	(1.00–1.38)	1.18	(1.00–1.39)
No	Ref		Ref		Ref		Ref		Ref		Ref	
**Females**												
Yes	1.32^h^	(1.11–1.57)	1.38^h^	(1.16–1.64)	1.32^h^	(1.11–1.57)	1.28^h^	(1.07–1.53)	1.28 ^h^	(1.07–1.53)	1.25^g^	(1.04–1.51)
No	Ref		Ref		Ref		Ref		Ref		Ref	

^a^Unadjusted; ^b^ adjusted for age; ^c^ adjusted for age, education; ^d^ adjusted for age, education, exposure to video display terminal (VDT) and health surveillance; ^e^ adjusted for age, education, exposure to VDT and health surveillance, exposure to work-related stress risk; ^f^ adjusted for age, education, exposure to VDT and health surveillance, exposure to work-related stress risk, diseases and chronic conditions; ^g^ = p < 0.05; ^h^ = p < 0.01

**Table 5:** Logistic regression analyses for overweight/obesity by night shifts, for men and women in a representative sample of 8000 Italian workers

Night shifts	Model 1 ^a^		Model 2 ^b^		Model 3 ^c^		Model 4 ^d^		Model 5 ^e^		Model 6 ^f^	
	OR (95% CI)		OR (95% CI)		OR (95% CI)		OR (95% CI)		OR (95% CI)		OR (95% CI)	
**Males**												
Never	Ref		Ref		Ref		Ref		Ref		Ref	
1 to 2 times/wk.	1.26	(0.86–1.54)	1.24	(0.85–1.81)	1.43	(0.97–2.12)	1.41	(0.94–2.10)	1.39	(0.93–2.08)	1.38	(0.92–2.07)
> 2 times/wk	1.08	(0.58–2.02)	1.04	(0.56–1.95)	1.05	(0.56–1.97)	1.04	(0.55–1.95)	1.03	(0.55–1.94)	0.99	(0.52–1.86)
**Females**												
Never	Ref		Ref		Ref		Ref		Ref		Ref	
1 to 2 times/wk.	1.45^g^	(1.02–2.07)	1.48^g^	(1.03–2.11)	1.59^g^	(1.11–2.28)	1.50^g^	(1.04–2.16)	1.49^g^	(1.03–2.16)	1.46^g^	(1.01–2.11)
> 2 times/wk	1.38	(0.80–2.40)	1.37	(0.79–2.39)	1.29	(0.74–2.26)	1.24	(0.71–2.17)	1.23	(0.70–2.17)	1.16	(0.66–2.04)

^a^Unadjusted; ^b^ adjusted for age; ^c^ adjusted for age, education; ^d^ adjusted for age, education, exposure to video display terminal (VDT) and

health surveillance; ^e^ adjusted for age, education, exposure to VDT and health surveillance, exposure to work-related stress risk; ^f^ adjusted for age, education, exposure to VDT and health surveillance, exposure to work-related stress risk, diseases and chronic conditions; ^g^ p < 0.05

Men working in the healthcare sector had a significantly larger Odds Ratio (OR 1.56, 95% CI 1.02–2.37) of being overweight or obese, but this lost significance after adjusting for confounders. Women working in agriculture or in other public and personal services were significantly associated with higher odds (4.4 and 2.8, respectively) of being overweight or obese, in both unadjusted and adjusted models. Adjustment for age and education (Model 3) raised the odds in transportation and healthcare (Table 2).

Male workers with permanent work contracts (Table 3) had a significantly higher likelihood of overweight or obesity (OR 1.52, 95% CI 1.23–1.88) than those with temporary contracts (Model 1), but the model was no longer significant after adjusting for age and education. A significant difference was seen again by adjusting for exposure to VDT and health surveillance (Model 4). However, women with permanent contracts were significantly associated with lower odds of overweight or obesity than those with temporary contracts, after adjusting for confounders.

Shift work gave interesting findings (Table 4). After adjusting for age, male shift workers had a significant difference - 1.25 times (95% CI 1.06–1.46) - in the likelihood of being overweight or obese. This association disappeared when including exposure to work-related stress risk and chronic conditions as confounders. There was a negative association with overweight or obesity also among female shift workers. In particular, These women were 1.32 times (95% CI 1.11–1.57) more likely to be overweight or obese than day workers, and the association remained when controlling for confounders.

Focusing on night shifts (Table 5), the likelihood of overweight or obesity was 1.5–1.6 times higher among women who worked 1–2 night shifts per week compared to day shifts.

No significant associations with overweight or obesity were found for occupational position, working hours and size of the firm.

## Discussion

The present study indicated that the risk of excessive fat accumulation in adipose tissue might be a consequence of exposure to several occupational or work-related factors and differs markedly in male and female workers. Previous studies observed sex differences in BMI levels and obesity prevalence in the same occupational group or with reference to specific occupational variables [20, 30, 31]. Although these data have been explained by calling into question demographic, socio-economic, and cultural or lifestyle factors, the common conclusion was that further research was needed on this topic. We obviously cannot exclude the possibility that our results related to sex differences may be partly due to determinants other than those we examined. However, the observation that in the adjusted analysis male and female workers showed different susceptibility to obesity, even after taking account of some key variables (age, education, sedentary work, psychosocial stressors and chronic diseases) suggests that, at least for some occupational factors, sex-specific differences have a significant role.

Therefore, assuming that this hypothesis is valid and considering workplaces as optimal and privileged adult settings where it is possible to plan and implement interventions to prevent and treat obesity, in our opinion these measures should certainly include - though not necessarily be limited to – promotion of healthy lifestyles. Regular physical activity and/or a healthy and balanced diet are general recommendations that are always valid for all people (or workers) regardless of sex, demographic and socio-cultural variables and type or characteristics of their work. Therefore, if we really want to take advantage of the workplace to work out more strategies to win the battle against obesity, we have to begin by considering that this condition is not just a consequence of a personal choice, but is caused more by a complex interplay between an individual and his or her environment [14]. To properly address this issue we need more qualitative and quantitative data to answer unsolved questions. For example, what are the occupational groups or worker categories that are at greater risk of obesity? Are there other occupational risk factors besides sedentary work and diet that may be associated with obesity? Are they modifiable? Considering the increase of women in the labor force and their growing involvement in roles and activities that were traditionally male-focused, might there be some gender differences in work-related factors potentially associated with obesity?

Our findings provide helpful information to tackle some aspects of these questions. First of all, analysing the main socio-demographic characteristics of the sample (sex, age and education), higher BMI and prevalence of overweight or obesity were observed in males, at older ages and in workers with lower education (Table 1). These results are in accordance with previous evidence, confirming that these variables are correlated with obesity [20–22, 30, 31, 38]. Therefore these socio-demographic determinants should be taken into account when trying to establish correlations between BMI and occupational groups or work-related factors, since they may help explain some differences in BMI [31].

The analysis of BMI in different occupational sectors indicated that male workers involved in healthcare and social assistance had the highest prevalence of overweight and obesity, while their female counterparts had higher prevalences in agriculture, fishing and hunting (overweight) and in construction, healthcare and social assistance (obesity) (Table 2). These data are in line with previous studies, further confirming that occupations requiring sedentary behavior or low levels of physical activity are marked by higher prevalences of overweight and obesity (although the association was weaker in women) [21, 30, 31, 38]. Nevertheless, there are some important differences since, differently from our results, several other groups reported higher prevalence rates in transportation and warehousing workers [20, 30–32, 38] and lower levels in the healthcare sector [32, 38]. In our study, using the ATECO classification, transportation and warehousing workers were included in the same group as information and communication workers, while in other studies workers in the healthcare and social assistance sector, with different jobs (e.g. healthcare practitioners and technical, healthcare support and protective services), were considered separately [32, 38]. Therefore, it is quite likely that these conflicting results are due to differences in classification of some occupational sectors although we cannot rule out an influence of socio-demographic and cultural factors [31].

With regard to socio-demographic characteristics, we also conducted an adjusted analysis to explore to what extent the BMI (and related overweight/obesity prevalence rates) in the occupational groups were affected by differences both in the distribution of these variables and other work-related factors (Table 2). The findings confirmed that, for female workers, these determinants could have a significant impact, changing the OR in specific occupational groups. On the other hand, few occupational categories (agriculture, fishing and hunting and other public and personal services) were associated significantly with increased OR even after adjustment, thus suggesting that other work-related factors, specific for these occupational groups and not captured or assessed in the present study, can contribute to overweight and obesity.

In addition, the differences in occupational effects by gender indicate that sex-specific factors other than socio-demographic and work-related determinants may influence the likelihood of overweight and obesity. Therefore additional research investigating the reasons for weight disparity between male and female workers in different job categories is much needed [20, 31].

Besides knowing the obesity prevalence rates for the different worker categories, it is also important to understand the reasons for them, in other words to identify the work-related factors (besides sedentary work, jobs with low physical demand, nutrition at work) that could possibly be associated with obesity. Subjects who worked long hours had a high risk of overweight/obesity [35, 43]; similarly, working ≥35 and > 40 or > 50 h per week was significantly associated with increased BMI in men [41] and with obesity in workers of both sexes [22, 38], whereas Kim et al. [36] found an association between this condition and long working hours only in women workers. We found no significant association between long working hours and overweight or obesity, even though women who worked > 40 and ≥ 55 h/week had the highest prevalence of overweight and obesity, respectively. Surprisingly, the highest level of obesity was seen among the men with the lowest working hours.

Shift workers had higher prevalence rates for overweight and obesity and the difference was significant for females. Luckhaupt et al. [38] and Di Milia and Mummery [35] obtained similar results, reporting higher prevalences of obesity and BMI levels in shift workers on night or rotating shifts compared to those on day or evening shifts. The increased odds of overweight/obese in female workers persisted after adjustment for socio-demographic characteristics, variables related to health and safety protection and chronic diseases (Table 4) and the trend was similar when taking night shifts (1 or 2 times per week) into account (Table 5). However, male shift workers were associated with an increased OR only when considering age, education or health surveillance and there was no significant association with night shift work (Tables 4 and 5).

These findings suggest that the relations among shift, night work and obesity is influenced by gender-specific variables. It has been suggested that inadequate or difficult working conditions can trigger a stress response that in turn may enhance the risk of obesity [44]. Indeed, when a person experiences a stressful condition the production of hormonal factors (especially of adipokines, which are strongly linked to appetite and fat storage) changes substantially [45–47]. Since the key sex differences in fat storage in men and women include different insulin sensitivity and adipokine production it is plausible to hypothesize that the gender-specific differences observed in our study are due to this sex asymmetry [13, 18, 19].

Then, assuming that stressors play a role in weight gain, few studies have investigated the association between certain psychosocial working conditions and obesity [21, 38]. In this regard, a correlation has been reported between hostile work environment and, to a lesser extent, job insecurity [38], whereas in the study by Choi et al. [21] job demand, supervisor and/or co-worker support were not associated with increased obesity prevalence and only low job control in female workers showed a significant difference. Our data are similar to those of Choi et al. [21] as no association was established between male and female workers’ exposure to work-related stress and prevalence rates for overweight and obesity.

We also studied the type of contract (permanent or temporary) as a possible work-related stress factor since a temporary contract is a major source of concern about becoming unemployed. This variable was taken into consideration by Luckhaupt et al. [38] who, while noting a higher prevalence of obesity among temporary workers compared to permanent ones, failed to identify any significant relation between boundary work and obesity. Similarly, the present study found no overall differences as regards the type of contract. However, the logistic regression models provided interesting findings highlighting important differences between male and female workers (Table 3). For women permanent contract seemed to serve as a protective factor against the risk of obesity, whereas this variable was associated with a higher OR in men.

Explaining these conflicting results is challenging since several other social, cultural and work-related factors (that were not analysed here) could be responsible for the differences. Nevertheless, once again, the divergent results for the two sexes suggests the need to focus primarily on the role of biological/physiological sex differences in facilitating or combating obesity in the workplace. Further research should verify whether exposure to specific occupational risk factors (long working hours, shifts and night work, psychosocial stressors) can influence - and how - the expression of these biological and physiological characteristics, or the functioning of some organ systems (e.g. the endocrine system) that could therefore explain the different propensities to obesity in male and female workers, because of the significant impact on metabolism and adipose tissue storage.

Finally, our data showed a significant association between overweight and obesity prevalence with several chronic conditions such as musculoskeletal, respiratory and cardiovascular diseases, both in males and females (Table 1). These results further underline the importance of preventing and treating obesity as excess weight gain is an important risk factor for several non-communicable diseases.

### Strengths, limitations and future perspectives

The study has some strengths. First, it addresses a large representative sample of the Italian working population, filling the gap of the lack of studies on occupational factors and risk of obesity in this country. Studies on national representative samples add value when investigating overweight and obesity, since there is ample evidence of external socio-cultural factors, such as diet, culture, acceptable lifestyles, and behavioral patterns affecting a person’s weight.

Secondly, we included several occupational factors in the risk of obesity by adjusting for main confounders. Some studies have looked at occupational aspects, but they mostly refer to specific occupational populations and sectors. Moreover, we considered a large set of occupational variables including some that are generally less investigated (e.g. work shifts, night work, type of contract) in relation to obesity, and relevant from the perspective of gender differences.

Finally, this study is not limited to checking for gender, but takes into consideration gender-specific differences in studying occupational factors associated with the risk of obesity in order to characterize the risk of obesity categories better and help identify workplace-targeted intervention strategies.

Some limitations must be addressed too with a view to future improvements. First, the cross-sectional design allows us to describe associations but not causation. In other words, we cannot draw causal inferences about the effects of the different variables on overweight and obesity since we cannot define the direction of the associations. However, data were collected as part of a national project, INSuLa, a well-established worker population survey, on a representative sample and included reliable information on several socio-demographic variables and working conditions.

This survey is now becoming a monitoring system to follow changes over time. The data collected in the study also gave some suggestions on how to integrate useful measures in the next waves attitudes and behaviors related to meals at work and physical activity will be considered in the future and linked to the BMI.

As second limitation is related to the self-reporting of body weight and height. Consequently BMI calculations are subject to error and our findings might be vulnerable to reporting bias. People have a tendency to overestimate their height and underestimate their weight and this self-reporting bias is stronger among overweight and obese individuals [48, 49]. However, in adults measured and perceived BMI are strongly correlated [50] and a limited number of studies analysing the differences between self-reported and measured anthropometrics in selected working categories have provided evidence that self-reported weight and height information is a reliable tool to assess BMI in large worker samples [51–53]. Future studies might investigate the validity of self-reported as opposed to measured BMI in specific Italian occupational groups, considering some sociodemographic differences such as sex, age, and education).

Finally, the study is based on a standardised questionnaire to survey Italian workers on their perception of health and safety at work. Even though we examined a broad set of factors related to work that can affect the BMI, some factors such as life satisfaction, job satisfaction, or income could not be investigated. These factors were not present in the standardised questionnaire aiming to provide a broad overview of different factors related to work and health, but might usefully be considered in the future in the light of our findings.

## Conclusions

Previous studies investigating the relationship between work and obesity found that BMI of workers may be significantly related to some work-related factors and also reported different obesity prevalence rates in various occupational groups and/or sectors. Several authors have therefore rightly suggested the possibility of specific measures to counteract obesity in the workplaces. These targeted intervention strategies should be particularly aimed at workers in job categories at high risk of obesity or exposed to occupational factors identified as possible elements facilitating this condition. Unfortunately, the effectiveness of such interventions is still unclear and uncertain. From a public health perspective, although there have been some encouraging signs of improvement the identification and promotion of up-to-date policies are yet under investigation (including food package labelling, public campaigns to boost people’s awareness, and health promotion, pricing and fiscal measures and changes in portion sizes), specifically with a view to reducing obesity [54–57]. So far no single country has achieved a significant and sustained decrease in obesity through the application of comprehensive and multidisciplinary policies [4, 11, 58].

The current study adds some interesting information to current knowledge on this topic, suggesting that gender differences need to be properly taken into account in any evaluation of the complex and multifaceted interactions between obesity and workers, work environment and/or working activities. This is an important and innovative aspect in this research area since our results indicate that the same obesogenic setting – the workplace - or more precisely the same occupational risk factors present in a work environment, may affect weight gain in men and women workers in substantially different ways. This perspective opens up interesting scenarios concerning the design and application of innovative strategies to tackle obesity in workplaces and other life settings, especially since currently gender differences and the related occupational risk factors are largely underestimated in the debate on the global obesity pandemic and therefore also in possible policies and solutions. Having to hand separate prevalence rates for male and female workers, together with knowledge of the possible sex-specific effects of some occupational risk factors, we can frame the issue of obesity in a more detailed and comprehensive way. This information would enable policy-makers to devise and implement programs and strategies to tackle obesity that would be surely more effective and specific.

In our opinion, the cornerstone of an efficient obesity prevention system should include a gender-tailored communication strategy. We agree with Kanter and Caballero [59, 60] that the same information (on obesity prevention) if conveyed in a gender-specific manner to male and female workers could achieve much better results. For example, particular attention should be paid to this communication aspect that takes account of gender differences, in combining public health actions in prevention packages. The incorporation of single actions and/or tools into broader coherent prevention strategies, targeting different population groups, might give better results at population level. However, at the same time it should be borne in mind that if these strategies are communicated too generically, without taking account of the specificities of individual groups, they inevitably lose effectiveness.

## Supplementary information

**Additional file 1.** Additional Materials 1. Tables on socio-demographic and occupational characteristics of the study sample. Tables show frequencies (numbers and percentages) about socio-demographic and occupational characteristics of the 8000 Italian workers sampled in the study.

## Data Availability

The data that support the findings of this study are available from INAIL but restrictions apply to the availability of these data, which were used under license for the current study, and so are not publicly available. Data are however available from the authors upon reasonable request and with permission of INAIL.

## Abbreviations

WHO: World Health Organization
BMI: Body mass index
INAIL: Italian workers compensation authority
ISTAT: Italian Statistics Institute
CATI: Computer-assisted telephone interviewing
ATECO: Economic activities
VDT: Video display terminal
OR: Odds ratio
CI: Confidence interval

## Footnotes

1 http://www.euro.who.int/en/health-topics/disease-prevention/nutrition/a-healthy-lifestyle/body-mass-index-bmi

## References

[1] Garrow JS (1988). Obesity and related diseases. 2nd rev. ed.

[2] World Health Organization (WHO). Obesity: preventing and managing the global epidemic, Technical report series: World Health Organization; 2000. p. 894. https://www.who.int/nutrition/publications/obesity/WHO_TRS_894/en/. Accessed 02 July 2019. 11234459

[3] Ackerman SE, Blackburn OA, Marchildon F, Cohen P (2017). Insights into the link between obesity and cancer. Curr Obes Rep.

[4] Afshin A, Forouzanfar MH, Reitsma MB, Sur P, Estep K, Lee A (2017). Health effects of overweight and obesity in 195 countries over 25 years. N Engl J Med.

[5] Camilleri M, Malhi H, Acosta A (2017). Gastrointestinal complications of obesity. Gastroenterology..

[6] Flegal KM, Kit BK, Orpana H, Graubard BI (2013). Association of all-cause mortality with overweight and obesity using standard body mass index categories: a systematic review and meta-analysis. JAMA..

[7] Koliaki C, Liatis S, Kokkinos A (2019). Obesity and cardiovascular disease: revisiting an old relationship. Metabolism.

[8] Riobó SP (2013). Obesity and diabetes. Nutr Hosp.

[9] Xu H, Cupples LA, Stokes A, Liu CT (2018). Association of Obesity with mortality over 24 years of weight history: findings from the Framingham heart study. JAMA Netw Open.

[10] Friedrich MJ (2017). Global obesity epidemic worsening. JAMA..

[11] Seidell JC, Halberstadt J (2015). The global burden of obesity and the challenges of prevention. Ann Nutr Metab.

[12] Global Health Observatory (GHO) data: Overweight and obesity. World Health Organization, 216. https://www.who.int. Accessed 20 Feb 2019.

[13] Mauvais-Jarvis F (2015). Sex differences in metabolic homeostasis, diabetes, and obesity. Biol Sex Differ.

[14] Blüher M (2019). Obesity: global epidemiology and pathogenesis. Nat Rev Endocrinol.

[15] Ng M, Fleming T, Robinson M, Thomson B, Graetz N, Margono C (2014). Global, regional, and national prevalence of overweight and obesity in children and adults during 1980–2013: a systematic analysis for the global burden of disease study 2013. Lancet (London, England).

[16] Organization for Economic Co-operation and Development (OECD) (2019). The heavy burden of obesity: the economics of prevention. Country note to the report the heavy burden of obesity: Italy.

[17] Sharma AM, Padwal R (2010). Obesity is a sign - over-eating is a symptom: an aetiological framework for the assessment and management of obesity. Obes Rev.

[18] Link JC, Reue K (2017). Genetic basis for sex differences in obesity and lipid metabolism. Annu Rev Nutr.

[19] Reue K (2017). Sex differences in obesity: X chromosome dosage as a risk factor for increased food intake, adiposity and co-morbidities. Physiol Behav.

[20] Birdsey J, Sussell AL (2017). Prevalence of obesity, no leisure-time physical activity, and short sleep duration among occupational groups in 29 states. J Occup Environ Med.

[21] Choi B, Schnall PL, Yang H, Dobson M, Landsbergis P, Israel L (2010). Sedentary work, low physical job demand, and obesity in US workers. Am J Ind Med.

[22] Park S, Pan L, Lankford T (2014). Relationship between employment characteristics and obesity among employed U.S. adults. Am J Health Promot.

[23] Brown WJ, Miller YD, Miller R (2003). Sitting time and work patterns as indicators of overweight and obesity in Australian adults. Int J Obes Relat Metab Disord.

[24] Freeman RB (2007). America works: the exceptional U.S. labor market.

[25] Persechino B, Fontana L, Buresti G, Rondinone BM, Laurano P, Imbriani M (2016). Professional activity, information demands, training and updating needs of occupational medicine physicians in Italy: national survey. Int J Occup Med Environ Health.

[26] Autor DH, Levy F, Murnane RJ (2003). The skill content of recent technological change: an empirical exploration. Quart J Econ.

[27] Lakdawalla D, Philipson T (2007). Labor supply and weight. J Human Res.

[28] Brownson RC, Boehmer TK, Luke DA (2005). Declining rates of physical activity in the United States: what are the contributors?. Annu Rev Public Health.

[29] Leso V, Fontana L, Iavicoli I (2018). The occupational health and safety dimension of industry 4.0. Med Lav.

[30] Allman-Farinelli MA, Chey T, Merom D, Bauman AE (2010). Occupational risk of overweight and obesity: an analysis of the Australian health survey. J Occup Med Toxicol.

[31] Proper KI, Hildebrandt VH (2010). Overweight and obesity among Dutch workers: differences between occupational groups and sectors. Int Arch Occup Environ Health.

[32] Caban AJ, Lee DJ, Fleming LE, Gómez-Marín O, LeBlanc W, Pitman T (2005). Obesity in US workers: the National Health Interview Survey, 1986 to 2002. Am J Public Health.

[33] Barlin H, Mercan MA (2016). Occupation and obesity: effect of working hours on obesity by occupation groups. Appl Econ Finance.

[34] Caruso CC (2014). Negative impacts of shiftwork and long work hours. Rehabil Nurs.

[35] Di Milia L, Mummery K (2009). The association between job related factors, short sleep and obesity. Ind Health.

[36] Kim BM, Lee BE, Park HS, Kim YJ, Suh YJ, Kim JY (2016). Long working hours and overweight and obesity in working adults. Ann Occup Environ Med.

[37] Ko GT, Chan JC, Chan AW, Wong PT, Hui SS, Tong SD (2007). Association between sleeping hours, working hours and obesity in Hong Kong Chinese: the 'better health for better Hong Kong' health promotion campaign. Int J Obes.

[38] Luckhaupt SE, Cohen MA, Li J, Calvert GM (2014). Prevalence of obesity among U.S. workers and associations with occupational factors. Am J Prev Med.

[39] Solovieva S, Lallukka T, Virtanen M, Viikari-Juntura E (2013). Psychosocial factors at work, long work hours, and obesity: a systematic review. Scand J Work Environ Health.

[40] INAIL (2014). Indagine nazionale sulla salute e sicurezza Dei lavoratori - Lavoratori e datori di lavoro.

[41] Gagliardi D, Di Tecco C, Ronchetti M, Bonafede AS, Corfiati M, Manca S, Russo S, Iavicoli S (2014). Progetto INSuLa: l’indagine conoscitiva sui Datori di Lavoro. G Ital Med Lav Ergon.

[42] Dragano N, Barbaranelli C, Reuter M, Wahrendorf M, Wright B, Ronchetti M, et al. Young workers’ access to and awareness of occupational safety and health services: age differences and possible drivers in a large survey of employees in Italy. Int J Environ Res Public Health. 2018;15(7). 10.3390/ijerph15071511. 10.3390/ijerph15071511 PMC6069130 30018272

[43] Shields M (1999). Long working hours and health. Health Rep.

[44] Porter JS, Bean MK, Gerke CK, Stern M (2010). Psychosocial factors and perspectives on weight gain and barriers to weight loss among adolescents enrolled in obesity treatment. J Clin Psychol Med Settings.

[45] Chrousos GP (2000). The role of stress and the hypothalamic-pituitary-adrenal axis in the pathogenesis of the metabolic syndrome: neuro-endocrine and target tissue-related causes. Int J Obes Relat Metab Disord.

[46] Chaput JP, Després JP, Bouchard C, Tremblay A (2007). Short sleep duration is associated with reduced leptin levels and increased adiposity: results from the Quebec family study. Obesity (Silver Spring).

[47] Forbes S, Bui S, Robinson BR, Hochgeschwender U, Brennan MB (2001). Integrated control of appetite and fat metabolism by the leptin-proopiomelanocortin pathway. Proc Natl Acad Sci U S A.

[48] Maukonen M, Männistö S, Tolonen H (2018). A comparison of measured versus self-reported anthropometrics for assessing obesity in adults: a literature review. Scand J Public Health.

[49] Connor Gorber S, Tremblay M, Moher D, Gorber B (2007). A comparison of direct vs. self-report measures for assessing height, weight and body mass index: a systematic review. Obes Rev.

[50] McAdams MA, Van Dam RM, Hu FB (2007). Comparison of self-reported and measured BMI as correlates of disease markers in US adults. Obesity (Silver Spring).

[51] Fonseca Mde J, Faerstein E, Chor D, Lopes CS (2004). Validity of self-reported weight and height and the body mass index within the "Pró-saúde" study. Rev Saude Publica.

[52] Korpela K, Roos E, Lallukka T, Rahkonen O, Lahelma E, Laaksonen M (2013). Different measures of body weight as predictors of sickness absence. Scand J Public Health.

[53] Wada K, Tamakoshi K, Tsunekawa T, Otsuka R, Zhang H, Murata C (2005). Validity of self-reported height and weight in a Japanese workplace population. Int J Obes.

[54] Cameron AJ, Zimmet PZ (2014). What will it take to curb the rise in obesity?. Med J Aust.

[55] Hawkes C, Smith TG, Jewell J, Wardle J, Hammond RA, Friel S (2015). Smart food policies for obesity prevention. Lancet..

[56] Organisation for Economic Co-operation and Development (OECD) (2017). Obesity update 2017. OECD public health topics, Paris, France.

[57] Wise J (2014). Open letter calls for global treaty to tackle obesity. BMJ..

[58] Rutter H (2018). The complex systems challenge of obesity. Clin Chem.

[59] Kanter R, Caballero B (2012). Global gender disparities in obesity: a review. Adv Nutr.

[60] Ostry AS, Radi S, Louie AM, LaMontagne AD (2006). Psychosocial and other working conditions in relation to body mass index in a representative sample of Australian workers. BMC Public Health.

