# Supplementary material for: Gender differences and occupational factors for the risk of obesity in the Italian working population
Source: BMC Public Health. 2020 May 16;20:706. doi: 10.1186/s12889-020-08817-z (PMC7229582; doi:10.1186/s12889-020-08817-z)
Supplement: Supplementary file 1 — Additional file 1. Additional Materials 1. Tables on socio-demographic and occupational characteristics of the study sample. Tables show frequencies (numbers and percentages) about socio-demographic and occupational characteristics of the 8000 Italian workers sampled in the study. [file 12889_2020_8817_MOESM1_ESM.docx]

**Tables on socio-demographic and occupational characteristics of the study sample**

| *Table 6. Socio-demographic characteristics of the study sample* | | |
| --- | --- | --- |
| Socio-demographics characteristics | ,N | (%) |
| Age groups | | |
| 16-24 | 425 | (5.3) |
| 25-34 | 1,649 | (20.6) |
| 35-44 | 2,491 | (31.1) |
| 45-54 | 2,361 | (29.5) |
| 55-64 | 1,074 | (13.4) |
| Gender | | |
| Male | 4,305 | (53.8) |
| Female | 3,695 | (46.2) |
| Educational level | | |
| < High school graduate | 2,461 | (31.2) |
| High school graduate | 3,893 | (49.3) |
| Graduate and post graduate | 1,538 | (19.5) |

| *Table 7. Occupational characteristics of the study sample* | | |
| --- | --- | --- |
| Occupational characteristics | N | (%) |
| Occupational sector | | |
| Agriculture, fishing, and hunting | 186 | (2.3) |
| Manufacturing/Primary industry/Mining/Utilities | 1,856 | (23.2) |
| Construction | 444 | (5.6) |
| Wholesale and retail trade/Automotive and motorcycle repair/Accommodation and food services | 1,413 | (17.7) |
| Transportation and warehousing/Information and communication | 634 | (7.9) |
| Professional, financial and business services | 938 | (11.7) |
| Healthcare and social assistance | 698 | (8.7) |
| Education services/Public administration, social security | 1,193 | (14.9) |
| Other public and personal services | 638 | (8.0) |
| Occupational position | | |
| Top and middle manager | 714 | (8.9) |
| White collar | 3,277 | (41.0) |
| Blue collar | 3,744 | (46.8) |
| Apprentice or other type of employment | 265 | (3.3) |
| Type of contract | | |
| Permanent job/work contract | 6,744 | (84.8) |
| Temporary job/work contract | 1,208 | (15.2) |
| Shift work | | |
| Yes | 2,674 | (33.4) |
| No | 5,326 | (66.6) |
| Night shifts | | |
| Never | 7,323 | (91.5) |
| 1 to 2 times/week | 414 | (5.2) |
| >2 times/week | 264 | (3.3) |
| Working hours | | |
| 1 -34 hours/week | 1,859 | (23.2) |
| 35 - 40 hours/week | 5,036 | (63.0) |
| 41 - 48 hours/week | 676 | (8.4) |
| 49 -54 hours/week | 261 | (3.3) |
| >=55h hours/week | 168 | (2.1) |
| Firm size | | |
| 1-9 | 1,316 | (16.5) |
| 10-49 | 1,614 | (20.2) |
| 50-249 | 1,687 | (21.1) |
| ≥ 250 | 3,019 | (37.7) |
| Health surveillance | | |
| Yes | 5,422 | (67.8) |
| No | 731 | (9.1) |
| Exposure to VDT ^a^ | | |
| Yes | 7,208 | (90.1) |
| No | 792 | (9.9) |
| Exposure to work-related stress risk | | |
| Yes | 4,436 | (55.4) |
| No | 3,564 | (44.6) |
| ^a^ Video terminal display | | |
